# Applying Exploratory Structural Equation Modeling to Examine the Student-Teacher Relationship Scale in a Representative Greek Sample

**DOI:** 10.3389/fpsyg.2018.00733

**Published:** 2018-05-15

**Authors:** Nikolaos Tsigilis, Athanasios Gregoriadis, Vasilis Grammatikopoulos, Evridiki Zachopoulou

**Affiliations:** ^1^Department of Journalism and Mass Media Communication, Aristotle University of Thessaloniki, Thessaloniki, Greece; ^2^Department of Early Childhood Education, Aristotle University of Thessaloniki, Thessaloniki, Greece; ^3^Department of Early Childhood Education, University of Crete, Rethymno, Greece; ^4^Department of Early Childhood Care, Alexander Technological Educational Institute of Thessaloniki, Thessaloniki, Greece

**Keywords:** exploratory structural equation modeling, early childhood education, teacher-child relationships, student-teacher relationship scale, measurement invariance

## Abstract

Teacher-child relationships in early childhood are a fundamental prerequisite for children's social, emotional, and academic development. The Student-Teacher Relationship Scale (STRS) is one of the most widely accepted and used instruments that evaluate the quality of teacher-child relationships. STRS is a 28-item questionnaire that assess three relational dimensions, Closeness, Conflict, and Dependency. The relevant literature has shown a pattern regarding the difficulty to support the STRS factor structure with CFA, while it is well-documented with EFA. Recently, a new statistical technique was proposed to combine the best of the CFA and EFA namely, the Exploratory Structural Equation Modeling (ESEM). The purpose of this study was (a) to examine the factor structure of the STRS in a Greek national sample. Toward this end, the ESEM framework was applied in order to overcome the limitations of EFA and CFA, (b) to confirm previous findings about the cultural influence in teacher-child relationship patterns, and (c) to examine the invariance of STRS across gender and age. Early educators from a representative Greek sample size of 535 child care and kindergarten centers completed the STRS for 4,158 children. CFA as well as ESEM procedures were implemented. Results showed that ESEM provided better fit to the data than CFA in both groups, supporting the argument that CFA is an overly restrictive approach in comparison to ESEM for the study of STRS. All primary loadings were statistically significant and were associated with their respective latent factors. Contrary to the existing literature conducted in USA and northern Europe, the association between Closeness and Dependency yielded a positive correlation. This finding is in line with previous studies conducted in Greece and confirm the existence of cultural differences in teacher-child relationships. In addition, findings supported the configural, metric, scalar, and variance/covariance equivalence of the STRS between males and females and between preschoolers (3–5 years) and early primary years (5–7 years). Latent factor means comparisons showed that females seem to have a warmer and more dependent relationship with their teachers and are less conflictual in comparison to males.

## Introduction

In her captivating TED talk in 2013, Rita Pierson, an educator with forty years of teaching experience mentioned that “children don't learn from people they don't like.” To further support this opinion, several studies have shown that the quality of teacher-child relationships is associated with smooth school adjustment and academic, behavioral and social-emotional success in school, especially for younger children (Baker, 2006; O'Connor and McCartney, 2007; Hughes et al., 2008; Murray et al., 2008a; Drugli and Hjemdal, 2013). For example, a warm and positive interaction with the teacher may promote a positive attitude toward engagement in the learning process (e.g., Baker, 2006; Drugli and Hjemdal, 2013) and make the child feel accepted and emotionally secure (Pianta, 1999; Hamre et al., 2014). Teacher-child relationships that are characterized by closeness (warm and affectionate interactions) have been shown to associate with effectiveness of task completion (Ahnert et al., 2013), with increased prosocial skills (Arbeau et al., 2010) and with better academic performance (Spilt et al., 2012). Overall, a high-quality teacher-child relationship enables the child to view the teacher as an important cognitive and emotional support (Mashburn and Pianta, 2006).

On the other hand, a negative teacher-child relationship can affect children's behaviors as well as their academic trajectories (Solheim et al., 2012). Children with less positive interactions with their teacher, often become disengaged or distant from classroom activities and may develop negative attitudes toward school (O'Connor, 2010; Cadima et al., 2015). Conflictual teacher-child interactions have often been associated with social withdrawal and antisocial behaviors (Murray et al., 2008b; Rudasill and Rimm-Kaufman, 2009) as well as with lower achievement in math and language skills (Hamre and Pianta, 2001; Pianta and Stuhlman, 2004b; Palermo et al., 2007; Doumen et al., 2008). In general, the relation among negative teacher-child relationships and children's conduct problems has been well-documented from various studies (e.g., Birch and Ladd, 1997; Buyse et al., 2009; Rudasill et al., 2010; Webb and Neurath-Pritchett, 2011).

An important issue that relates with the quality of teacher-child interactions is the consistency with which some children manage to maintain the quality of their relationships with their teachers. While some children succeed into forming positive relationships with their teachers, year after year, other seem to experience more variability in the quality of their relationships with them (Pianta and Stuhlman, 2004a). A part of this variation in the teacher-child relationship quality may be explained by specific children's characteristics such as age, gender, race and socioeconomic status (Jerome et al., 2009). Especially, the characteristics of gender and age are of particular interest for the current study.

When it comes to gender, there are studies that did not reveal any significant associations between children's gender and the quality of teacher-child relationships (e.g., Murray et al., 2008a). However, other studies (e.g., Koch, 2003; Ang et al., 2008) have shown that boys and girls receive differential treatment in the classroom. Teachers have been found to describe closer relationships with girls and more conflictual relationships with boys (Silver et al., 2005; Baker, 2006). In addition, teacher-child closeness has been found to be more predictive of school competence for girls than boys, and teacher-child conflict is described as a stronger predictor of aggressive behaviors for boys than girls (Ewing and Taylor, 2009). Hence, gender stereotyping in the classroom seems to continue to be an important parameter of the quality of teacher-child relationships (Glüer and Gregoriadis, 2017).

When it comes to the relation between teacher-child relationships and children's age, the available research findings are relatively limited. In her doctoral dissertation, Saft (1994) found that teachers reported more conflicts with younger children, while Saft and Pianta (2001) also reported that child age and ethnicity were consistently associated with teacher-child relationships. Similar findings were reported in another study (Pianta and Stuhlman, 2004a), where moderate correlations among teachers' ratings of conflict across years were found. If we add to this evidence the fact that affective and behavioral problems occur differently at different ages (Zill, 1999), then it is apparent that teachers' perceptions of relationships with students might vary predictably by age of student and that the relationship of age to teacher-child relationships requires further examination.

To summarize insofar, it seems that the quality of teacher-child relationships in daily classroom life is acknowledged as one of the most influential factors for an effective learning environment. Such an increasing recognition of the importance of teacher-child relationships for children's development and academic progress highlights even more the need for precise and accurate measurement of the quality of these relationships (Tsigilis et al., 2017).

### Measuring quality of teacher-child relationships

In the existing literature, there are some instruments available for evaluating the quality of teacher-child relationships in early childhood education, like for example the Child Appraisal of Relationship with Teacher Scale (Vervoort et al., 2015), the Classroom Assessment Scoring System (Pianta et al., 2008), the Student-Teacher Observation Measurement (Glüer and Hannover, 2012), the Young Children's Appraisals of Teacher Support (Mantzicopoulos and Neuharth-Pritchett, 2003), and the Student-Teacher Relationship Scale-STRS (Pianta, 2001). The STRS is the most widely accepted and used instrument to evaluate teachers' perceptions of their relationships with individual students (Gregoriadis and Grammatikopoulos, 2014) and it is the measure applied in this study.

STRS combines research on parent-child and teacher-child relationships with attachment theory (Pianta, 1999). The initial instrument was designed to measure teacher-child relationships with children from preschool to grade 3 (ages 4–8), but it has also been used in studies with older children (Koomen et al., 2012). The STRS includes 28 items (Pianta, 2001) which are rated on a 5-point Likert-type scale (from “definitely does not apply” to “definitely applies”). It contains three subscales that assess three relational dimensions, Closeness, Conflict and Dependency. The Closeness subscale evaluates positive affect and the degree of children's and teachers' personal communication (e.g., “I share an affectionate, warm relationship with this child”). The Conflict subscale includes items that show that the teacher and the child are frequently at odds with each other (e.g., “This child and I always seem to be struggling with each other”). The Dependency subscale assesses the level of inappropriate developmental dependency a child might have (e.g., “This child reacts strongly to separation from me”) (Pianta, 1999).

The STRS has been applied in numerous countries and different cultural contexts like Finland, U.K, Italy, Norway, Spain, Greece, Portugal, Turkey, Germany, and USA. The three-factor structure of the STRS and the 28-item solution has been confirmed in several occasions with children aged 4–8 years in Norway (Drugli and Hjemdal, 2013), Italy (Fraire et al., 2013), Netherlands (Spilt and Koomen, 2009), Greece (Gregoriadis and Tsigilis, 2008), Turkey (Koka, 2010), Sweden (Henricsson and Rydell, 2004), and in USA (e.g., Pianta, 2001; Saft and Pianta, 2001). However, there are several studies as well that have showed mixed findings regarding the original item solution and factor structure of the STRS, especially when analyzed with confirmatory factor analysis (CFA). Drugli and Hjemdal (2013) used CFA to test the original factor structure of the STRS in a Norwegian national sample. Their findings did not confirm the proposed three-factor structure and subsequently they focused their analysis on the Short Form of STRS. In a US study, Webb and Neurath-Pritchett (2011) applied CFA in a sample of 445 children. Their results did not support the original item structure and their modified model included a 26-item solution. Similarly, Solheim et al. (2012) did not find an adequate model fit, when testing the original three dimensional STRS model using CFA. Instead, they proposed a modified 25-item 3-factor version that displayed an acceptable fit. A more recent study in Germany and Austria (Milatz et al., 2014) also run CFA in a large sample and their findings did not support the STRS factor structure. Subsequent exploratory factor analyses (EFA) resulted in major item reductions in that study, followed by further CFAs on validation samples. In more details, their modified STRS model included an adjusted 3-factor structure with 12 items. EFA analyses also showed good model fit in three other studies in Greece (Gregoriadis and Tsigilis, 2008), in Italy (Fraire et al., 2013) and in Germany (Glüer and Gregoriadis, 2017) resulting in a 26-item, a 22-item, and a 28-item solution, respectively. In addition, in their effort to confirm the factor structure of the STRS, two studies adopted an item-parceling approach. In the first study, Tsigilis et al. (2017) applied a CFA in a Greek sample without finding an adequate fit and proceeded to run an EFA, where again they reported mixed findings and item cross-loadings. Finally, they run a CFA with an item-parceling approach resulting in a model with a very satisfactory fit. The second study (Cadima et al., 2015), also applied both EFA and CFA in Portuguese and Belgian students without satisfactory results. Subsequently, they conducted CFA with item-parceling approach which showed good fit.

To summarize, a large portion of the relevant literature shows a repeating pattern regarding the difficulty to confirm the STRS original factor structure with CFA, while it is supported relatively well with EFA. As Tsigilis et al. (2017) already mentioned, “the findings from all these studies on the STRS imply that the factorial validity of the STRS may vary, and highlight the need for further exploration of the validity of STRS …” (p. 3).

A restricted number of studies examined an important psychometric property of the STRS that is whether the scale has the same meaning and functions equivalently among various groups (Tsigilis and Gregoriadis, 2008; Webb and Neurath-Pritchett, 2011; Koomen et al., 2012; Milatz et al., 2014; Cadima et al., 2015). These authors mainly focused on measurement invariance across gender, age, and ethnic groups, with mixed results. For example, Milatz et al. (2014) found strong invariance between boys and girls in a German sample, whereas Koomen et al. (2012) reported only weak invariance in a Dutch sample. With regard to the age effect, findings showed partial strong factorial invariance across kindergarteners, first graders, and second graders (Milatz et al., 2014). On the other hand, Koomen et al. (2012) found only partial weak invariance between a wider span of age (8–12 years old). Additional research activity is needed to better understand whether teachers' perceptions about their relationships with students differentiate in relation to pupil's gender and age. Moreover, given that the associations among the three STRS dimensions are fluctuated, studies should be extended and examine the invariance of the structural parts of the scale (e.g., correlations among latent factors).

#### A new approach for scale assessment: exploratory structural equation modeling (ESEM)

The above mentioned literature review showed that the factorial structure of the STRS was examined using two multivariate statistical techniques, namely EFA and CFA. EFA is usually applied at the early stages of an instrument's development, during which no specifications are made in regard to its structure. That is why some authors characterize it as data-driven technique (Brown, 2015). Its purpose is to achieve a simple and interpretable solution. However, EFA has several limitations. First, EFA cannot incorporate methods effect adjustments. For example, it is not unusual to have two items with strong similarities in the wording. Consequently their covariance cannot be explained merely from their relation to their latent constructs and a residual correlation usually needs to be added. Second researchers are often interested in comparing the scores of an instrument across different groups (e.g., gender, ethnicity) or across time. This comparison is meaningful only if the same score has the same interpretation for the different groups. Implementation of EFA is incapable of formally testing the equivalence of an instrument's structure among different populations or time.

On the other hand, in CFA the structure of an instrument (e.g., number of underlying factor, items-factors relationships) is defined a priori based on theoretical assumptions and/or previous EFA results. Thus, CFA is considered a theoretically driven approach. In a typical CFA a simple structure is specified, in which items are related to only one latent factor, constraining loadings to other factors to zero. Moreover, correlated uniqueness can be included in the model. An important advantage of CFA is its ability to determine the degree to which measurement model generalizes across groups or across time, which subsequently enables comparison of obtained scores.

However, in applied research it is hard to find a well-fitted model according to the existing pre-specified criteria without any additional modifications. This is especially true for large instruments, with multiple latent factors, each assessed by a reasonable number of items (Marsh, 2007). In an attempt to improve model's fit, researchers frequently shift to an exploratory mode by introducing certain modifications based on the examination of the ill fit aspects of the model. Part of the frequently poor model fit lies in the overly restricted assumption of zero cross-loadings (Asparouhov and Muthén, 2009; Marsh et al., 2014). Several authors admit that in the behavioral sciences it is difficult to develop items that assess only one aspect of the construct. “A measurement instrument often has many small cross-loadings that are well-motivated by either substantive theory or by the formulation of the measurements” (Asparouhov and Muthén, 2009, p. 398). Another side effect of ignoring the cross-loadings may lead to inflated associations among latent factors, threatening the instrument's discriminant validity. This happens because when non-zero cross-loadings are fixed to zero, the correlation between factor indicators representing different factors is forced to go through their main factors only.

Motivated by the above shortcomings of both EFA and CFA, Asparouhov and Muthén (2009) developed a new approach, namely the Exploratory Structural Equation Modeling (ESEM). ESEM effectively combines the advantages of both EFA and CFA words. In particular, ESEM allows for less restrictive measurement models in which items load to all factors and with factor loading matrix rotation to obtain an interpretable solution. On the other hand, it gives access to the usual fit-indices and parameter estimation (e.g., alternative fit indices, standard errors, error/uniqueness variances). Simulation studies as well as empirical studies have shown that ESEM provides better fit to the data in relation to CFA and latent factors correlations are closer to true associations and to theoretical expectations (Asparouhov and Muthén, 2009; Guay et al., 2015; Chiorri et al., 2016). To the best of our knowledge no research used ESEM approach to study the factorial structure of the STRS. It is possible that the discrepancy between EFA and CFA solutions which noticed in the literature is due to the multiple cross-loadings which have not been appropriately modeled in the CFA. Thus ESEM seems to be a promising alternative to overcome EFA and CFA restrictions and reveal the factorial structure of the STRS.

#### Purpose of the study

The purpose of this study was threefold. The first purpose was to examine the factor structure of the STRS in a nationally representative sample in the Greek early childhood education system. Toward this end, the ESEM framework was applied in order to overcome the limitations of EFA and CFA. The second purpose attempted to confirm previous findings in the literature (e.g., Gregoriadis and Tsigilis, 2008) and offer a firm conclusion about the cultural influence of teacher-child relationship patterns in the Greek settings. The third purpose focused on the invariance of STRS across gender and across two age groups (preschoolers and early primary years children).

## Materials and methods

This study is a part of the Early-Q Thales project (code MIS379429). The Early-Q Thales project (2012–2015) was a project supported by a grant from the European Union (European Social Fund) and national resources under the operational program “Education and Lifelong Learning” (http://www.edulll.gr/wp-content/uploads/2012/08/APOF_1H_379429_ADA.pdf - in Greek).

### Participants

A multistage sampling technique was applied in the current study, in order to collect a representative sample at a national level. Initially, two municipalities from each of the 12 educational districts of the Greek early childhood education system were randomly selected. Then, from each municipality a number of maximum 25 kindergarten and childcare centers were randomly selected. From each center, one classroom was again randomly selected to participate in the current study. Some municipalities had fewer than 25 units and thus the final amount of the classrooms participated in the current study totaled to 535 early childhood classrooms.

Five hundred and thirty-five early childhood classrooms participated in the study, 338 (63.2%) of which were public kindergarten classrooms and 197 (36.8%) municipal childcare classrooms. Also, 535 early childhood teachers (338 kindergarten teachers and 197 early educators) participated in the study with a mean age 42.98 (*SD* = 7.23) and a mean of 16.52 (*SD* = 7.83) of teaching years. The vast majority of teachers were female and only six of them were male (1.12%). Approximately 8 children (four boys and four girls) were randomly selected from each classroom and the teachers completed the measures for these children. For each classroom, only one teacher rated the children. In some classrooms, where the whole population was less than eight children, the total number of children participated in the study and the sample size reached the number of 4,158, from which 2,084 (50.1%) were boys and 2074 (49.9%) were girls. Their mean age was 4.93 years (*SD* = 0.93).

### Measures

The STRS version that was used in this study was the adapted STRS created by Koomen et al. (2012). The authors of that study attempted a revision of the STRS, by adding in the original 28-item version six additional items, three for Closeness and three for Dependency. Their purpose, among others, was to further validate the dimensionality of the STRS with CFA and to improve the scale, especially the measurement of Dependency subscale. The results showed an overall acceptable fit for the three-factor model. Koomen et al. (2012) resulted in a slightly changed 28-item version of the STRS with four of the original items (6, 9, 19, 21) being removed and two of the newly introduced (30, 31). Cronbach's alpha for Closeness was 0.88, for Conflict 0.90, and for Dependency 0.78 (Koomen et al., 2012). This is the reason why selecting the adapted version of the STRS for our study seemed to be more appropriate in terms of acceptable psychometric properties.

So, the measure used in the current study is the adapted STRS with 28 items. It includes three subscales, Closeness (11 items), Conflict (11 items), and Dependency (6 items). The items are rated by a five-point Likert scale from 1 (“definitely does not apply”) to 5 (“definitely applies”).

### Data collection

The data of this study were part of a larger data set collected for the Early-Q Thales project. Prior to data collection, the Greek ministry of education issued an official license to the project in order to have access to the participating early childhood classrooms. The directors of each educational district and the preschool centers principals were also informed about the purpose and the methodology of the project, and written consent forms were obtained. Additionally, all kindergarten teachers and early educators were informed about the study and accepted to participate in it. The assessors of the Thales project visited each classroom in a period of 1 month and randomly selected the children from each classroom. Then, the teachers were informed about the completion of the questionnaires for the specific children and were administered the STRS questionnaires, which they returned completed by the end of the day.

It should also be mentioned, that the current study did not require the direct participation of any student. However, the researchers sent a letter to the parents of all children from the participating early childhood classrooms informing them about the objectives of the study and describing that the teachers would complete a questionnaire for their children. The letter also mentioned that there was an official license from the Greek Ministry of Education for the implementation of this study. In addition, parents were informed that if they wanted the teacher not to complete the questionnaire for their child, they could ask the preschool center principal to exclude the child from the procedure. Finally, parents were given the researchers' contact information in case they wanted additional clarifications.

#### Statistical analysis

Confirmatory factor analysis as well as exploratory structural equation modeling were used to study the underlying structure of the STRS. All analyses were conducted using M*plus* ver. 7.3 (Muthén and Muthén, 2012).

#### Estimation method

The selection of the appropriate estimation method is an important decision for estimating the model parameters and testing the invariance of an instrument (Sass, 2011). Preliminary data analysis showed that the percent of missing values for each of the examined variables was extremely low ranging from 0.1 to 0.8%. These trivial percentages were not a threat for the analysis and this study used the M*plus* default options. Next, descriptive statistics were first calculated. Results showed statistically significant levels of skewness and kurtosis. Moreover, Mardia's coefficient for multivariate kurtosis was also statistically significant (Mardia's coefficient = 345.6, *p* < 0.001), suggesting that the data deviated from multivariate normality. Lastly and most important, teachers' responses to STRS were measured in an ordinal scale and thus linearity (i.e., linear relationships between variables) is difficult to be assumed (Bowen and Masa, 2015; Pendergast et al., 2017). Based on the above considerations and findings the mean and variance-adjusted Weighted Least Squares estimator (WLSMV) was employed as the most appropriate for these type of data (Sass, 2011; Bowen and Masa, 2015; Brown, 2015). When WLSMV is used, the polychoric correlation matrix is entered for analysis and adjusted chi-square value and robust standard errors are estimated. The polychoric correlation matrix combined with the WLSMV estimator can address the ordinality of the observed variables (Bowen and Masa, 2015). Simulation studies have consistently shown that WLSMV provides satisfactory results (Flora and Curran, 2004; Beauducel and Herzberg, 2006; DiStefano and Morgan, 2014). Brown (2015) characterized WLSMV as the best option for CFA modeling in the face of categorical data. It should be noted that no cell had zero frequency suggesting that polychoric correlation matrix could be reasonably accurately calculated (Pendergast et al., 2017).

By default, M*plus* uses a Full Information Maximum Likelihood (FIML) estimation approach to deal with missing values. FIML works effectively with raw data. When however the WLSMV estimator is selected, summary data are entered for analysis and the FIML cannot be employed. Instead, the listwise or pairwise deletion approach can be used. Asparouhov and Muthén (2010) showed that pairwise deletion works better that the listwise and is currently the default setting in M*plus* for handling missing values with the WLSMV estimator.

#### Multigroup analysis

Within the structural equation modeling framework, it is possible to test the invariance (or equivalence) of a postulated model across several groups. Examination of an instrument's invariance is a very important aspect of its psychometric properties. Demonstrating that an instrument has the same form and functions equivalently among multiple groups or occasions, allows researchers to: (a) compare groups latent factors mean scores, (b) infer about instruments' cross-cultural robustness, and (c) conduct longitudinal analysis (Tóth-Király et al., 2016; Guo et al., 2017; Pendergast et al., 2017). According to the proposed procedure for examining the invariance of a model, equality constraints are imposed on a particular set or all parameter estimates. Parameter constraints are imposed in a logically ordered and increasingly restrictive fashion (Byrne, 2012; Bowen and Masa, 2015). Marsh et al. (2014) presented a taxonomy of multigroup invariance test within the exploratory structural equation modeling framework. The present study examined the following types of STRS invariance: (a) configural invariance (equal form of factor structure among the groups, with no constraints), (b) weak invariance (equal unstandardized item loadings), (c) strong invariance (equal items thresholds), (d) invariance of factor variance-covariances and (e) invariance of factor means (Meredith, 1993).

In testing for configural invariance, the proposed factor structure of the examined instrument is fitted to all groups simultaneously. At this step no equality constraints are imposed on any of the model parameters. Demonstration of configural invariance is a necessary prerequisite for proceeding to measurement invariance. If configural invariance does not hold, further invariance testing is meaningless and the analysis is terminated. Weak or metric invariance postulates that items loading have the same unstandardized value for all groups. Establishment of weak invariance means that latent factors are measured in the same way across groups, enabling further examination of the invariance of factor variances and covariances. Equality of item loadings is the minimum necessary condition for considering measurement invariance of an instrument (Marsh et al., 2010).

Strong or scalar invariance assumes that intercepts or thresholds are invariant given the invariance of items loading. When observed variables are continuous, intercepts invariance is examined. On the other hand, if observed variables are dichotomous or ordered, categorical thresholds are estimated and their invariance is tested in an analogous to intercept invariance way. Thresholds of observed ordinal variables are conceptualized as categorizing underlying normality that distributes continuous variables (Savalei, 2011; Bowen and Masa, 2015). A threshold then is a point on the unobserved continuous distribution, where participants vary between two adjacent response categories. The number of thresholds is equal to the number of categories minus one. The demonstration of strong invariance mean that latent factors are measured on the same scale, and allows for their means to be compared (Meredith, 1993; Sass, 2011).

Although invariance of factor variance-covariances is not frequently examined, it is an important aspect of multidimensional measures (Marsh et al., 2010). This type of invariance examines distinctiveness of an instruments' dimensions across groups or occasions. A comparison of latent factor means requires partial or full scalar invariance and can be tested indirectly through selecting a group as the reference group, in which its mean and variance are set to 0 and 1, respectively.

#### Assessment of model fit and invariance

The usual way to estimate the fit of a model is with chi-square. Significant chi-square values denote a discrepancy between the observed and the implied covariance structure, thus rejecting the tenability of the examined model. However, this statistic suffers from several shortcomings, which are well-documented in the literature (e.g., requirement of the multivariate normality, effect of sample size). The most salient issue with chi-square is its sensitivity to sample size. Because the sample size is taken into account for chi-square calculation, the null hypothesis might be over rejected with large number of participants. Consequently, overreliance on the chi-square test may lead to the rejection of well-fitted models. In addition, the chi-square statistics leads to yes/no decision, while not knowing the degree of discrepancy between the observed and the implied covariance structure.

In order to address chi-square shortcomings, alternative fit statistics have been developed in an attempt to adjust for sample size and model complexity. Thus, the evaluation of a model's fit is frequently supplemented with various goodness-of-fit indices. The Comparative Fit Index (CFI) and the Root Mean Square Error of Approximation (RMSEA) are two widely used indices. These indices were selected because they are frequently used for model fit and model comparison in invariance testing and are provided by M*plus*, when using the WLSMV estimator (e.g., Sass, 2011; Pendergast et al., 2017). Despite the fact that alternative fit indices are appealing, there is disagreement as to what values suggest a good model fit and whether they should be used as “golden rules” (Marsh et al., 2004). Among the many goodness of fit indices examined in their simulation study, Hu and Bentler (1999) suggested a two-index presentation strategy comprising the RMSEA, adding one of the other examined indices, which includes CFI. These authors proposed a more rigorous value for a relative good model fit, which is CFI-value around 0.95 and RMSEA close to 0.06.

Examination of the invariance is conducted by comparison of the various models (e.g., configural model versus weak model). Because constraints are introduced in an increasing fashion, subsequent models are nested. It is well-known that, when models are nested, the χ^2^ difference between the two models (Δχ^2^) is itself χ^2^-distributed, with degrees of freedom equal to the corresponding difference in degrees of freedom. Non-significant values suggest that the addition of parameter constraints did not worsen the fit of the model and the specific invariance is acceptable. However, Δχ^2^ suffers from the same shortcomings as the χ^2^ statistic (Sass, 2011; Byrne, 2012; Pendergast et al., 2017). Thus, changes in CFI (ΔCFI) and RMSEA (ΔRMSEA) have also been proposed to supplement Δχ^2^. ΔCFI ≥ −0.01 (Cheung and Rensvold, 2002; Chen, 2007) and RMSEA ≤ 0.015 (Chen, 2007) are indicative of minor deterioration of the fit of the examined models suggesting that the specific invariance test holds. Given the relative large sample size of the present study, less emphasis was placed on χ^2^ and Δχ^2^ during the interpretation of a model's fit. A similar approach was adopted in previous studies that examined the factorial structure and measurement invariance of STRS (Koomen et al., 2012; Milatz et al., 2014).

## Results

### STRS factor structure—ESEM vs. CFA

Marsh et al. (2010, 2014) suggested that researchers should routinely examine the necessity of using ESEM by comparing its fit to CFA. If both approaches yield similar fit indices, then CFA should be preferred as more parsimonious. If, on the other hand, ESEM provides better fit than CFA, it means that the specification of no cross-loadings is indeed an overly restricted condition. So, CFA and ESEM were conducted and the results were compared in terms of goodness-of-fit indices and evaluation of parameter estimates (Marsh et al., 2010).

Goodness-of-fit indices showed that ESEM solution (χ^2^ = 4741.1, *df* = 297, CFI = 0.949, RMSEA = 0.060, 90%CI = 0.058–0.061) provided a far better fit to the data in relation to the CFA solution (χ^2^ = 12307.6, *df* = 347, CFI = 0.862, RMSEA = 0.091, 90%CI = 0.090–0.092). Given that the two models are nested, their comparison is meaningful (Asparouhov and Muthén, 2009). Results showed that the ESEM was substantially superior to the CFA model (Δχ^2^ = 4323.8, *df* = 50, *p* < 0.001, ΔCFI = +0.087, ΔRMSEA = −0.031). In addition, the RMSEA 90% confidence intervals did not overlap with each other. In both models the item loadings were positive and tended to range from modest (above 0.40) to high (>0.90) (Table 1). With regard to ESEM solution, all items loaded on the factors that were initially destined to measure (range 0.45–0.95, *M* = 0.68, *SD* = 0.14). The only exception from this pattern was noticed for item Closeness #12, which loaded primarily on the Dependency factor. An examination of the cross-loadings revealed that they are substantially lower (absolute mean value |M| = 0.14, *SD* = 0.12, max = 0.420, min = 0.002) than the primary loadings. These results clearly indicate that the CFA requirement of fixing the secondary loadings to 0 is excessively restrictive for the case of the STRS.

**Table 1:** STRS item loadings for the CFA and ESEM solution.

	**CFA**	**ESEM**		
		**F1**	**F2**	**F3**
CL1	0.789	**0.605**	−0.174	0.234
CL3	0.627	**0.494**	0.103	0.420
CL4	0.497	**0.436**	−0.139	0.011
CL5	0.811	**0.644**	−0.027	0.380
CL7	0.629	**0.540**	0.071	0.319
CL12	0.613	0.329	−0.134	**0.448**
CL15	0.589	**0.774**	0.201	−0.036
CL27	0.600	**0.839**	0.321	0.002
CL28	0.795	**0.703**	−0.122	0.120
CL32	0.640	**0.500**	0.040	0.376
CL34	0.764	**0.645**	−0.061	0.248
CO2	0.846	−0.013	**0.861**	−0.199
CO13	0.761	0.025	**0.788**	0.065
CO11	0.724	−0.027	**0.715**	0.077
CO16	0.760	−0.099	**0.704**	−0.034
CO18	0.877	0.074	**0.951**	−0.161
CO20	0.913	−0.026	**0.920**	−0.196
CO22	0.809	−0.027	**0.802**	0.055
CO23	0.829	−0.263	**0.647**	0.130
CO24	0.867	−0.412	**0.577**	0.045
CO25	0.602	−0.014	**0.588**	0.304
CO26	0.737	0.048	**0.795**	−0.115
DE8	0.721	−0.024	0.059	**0.719**
DE10	0.805	−0.055	0.002	**0.840**
DE14	0.574	−0.102	0.109	**0.613**
DE17	0.638	0.122	0.412	**0.482**
DE29	0.700	0.186	−0.078	**0.621**
DE33	0.666	0.009	0.104	**0.666**

The association between Closeness and Conflict was more pronounced in the ESEM solution (Table 2). On the other hand, correlation coefficients between Dependency and Closeness as well as between Dependency and Conflict were noticeably lower for the CFA solution. Based on the above findings, the ESEM model was selected as the more tenable for describing the STRS responses. In addition, the goodness-of-fit for the ESEM model showed the best fit in relation to previous research (e.g., Koomen et al., 2012; Milatz et al., 2014).

**Table 2:** Associations among the STSR dimensions.

	**Closeness**	**Conflict**	**Dependency**
Closeness	1.0	−0.522	0.190
Conflict	−0.490	1.0	0.181^*^
Dependency	0.514	0.279	1.0

*Below diagonal CFA solution, Above diagonal ESEM solution*.

Estimates of the subscales score reliability were calculated using omega coefficient (McDonald, 1999). Omega is based on the common variance and represents the ratio of the true-score variance to the total variance (ω = (Σ|λ_i_|)^2^/([Σ|λ_i_|]^2^ + Σδ_ii_), where λ_i_ are the standardized factor loadings, δ_ii_ the standardized item uniqueness and i subscript denotes a particular item of the scale). Contrary to alpha coefficient, omega does not require equal factor loadings or uncorrelated error variances (Dunn et al., 2014; Trizano-Hermosilla and Alvarado, 2016). As a result, ω takes into account the strength of the association between the indicators and the construct as well as the item specific measurement bias. Omega values were 0.888 for Closeness, 0.950 for Conflict, and 0.797 for Dependency, suggesting acceptable scale reliability.

### STRS invariance across gender

The first step before examining any type of invariance is to establish the best fitting model for each subgroup (Dimitrov, 2010; Byrne, 2012; Bowen and Masa, 2015; Pendergast et al., 2017). Thus, the ESEM model was run separately for boys and girls. Despite the fact that chi-square values were statistically significant, the approximate fit indices all suggested a good model fit for both groups (Table 3). Next, the configural invariance was tested by simultaneously fitting the ESEM model to both groups with no additional constraints. Based on the approximate fit indices the equal form of STRS structure across gender was supported. The following model examined the weak invariance (equal factor loading). The fit of the model to the data was satisfactory as it is indicated from the fit indexes. The chi-square difference between the two models was significant however, both ΔCFI and ΔRMSEA suggested that the fit of the model was improved. Thus, STRS's weak invariance was acceptable. Next, thresholds were constrained to be equal for boys and girls. When this invariance constraint was imposed, the fit remained satisfactory. Again, the chi-square difference was significant, but changes in CFI and RMSEA were trivial, indicating that the imposition of identical factor loading and thresholds did not impair the fit of the model. The above findings are encouraging and seem to support the measurement invariance of the STRS across students' gender. More importantly demonstrating measurement invariance enables the examination of the invariance of structural aspects of the STRS such as variance-covariances and latent means.

**Table 3:** Summary of goodness-of-fit statistics for the examined invariance across gender.

	**χ^2^**	***df***	**CFI**	**RMSEA**	**90%CI RMESEA**	**Δχ^2^ (*df*)**	**ΔCFI**	**ΔRMSEA**
Males	2421.6	297	0.956	0.059	0.057–0.061	–	–	–
Females	2330.6	297	0.942	0.057	0.055–0.059	–	–	–
Configural Invariance	4752.7	594	0.950	0.058	0.057–0.060	–	–	–
Metric Invariance	2679.2	669	0.976	0.038	0.037–0.040	121.8 (75)^**^	+0.026	−0.020
Scalar Invariance	3052.3	778	0.973	0.037	0.036–0.039	452.5 (109)^**^	−0.003	+0.001
Variance-Covariance Invariance	1942.8	784	0.986	0.027	0.025–0.028	6.42 (6)^ns^	+0.013	−0.01
Latent means invariance	3321.4	787	0.969	0.039	0.038–0.041	307.9 (3)^**^	−0.017	+0.012

** *p < 0.01*.

The next invariance model tests the equality of variance and covariance among the latent factors, assuming strong invariance. The chi-square change was not statistically significant and the ΔCFI as well as the ΔRMSEA suggested improvement of the fit, showing that the latent factor variance and covariance can be viewed as invariant across gender. Interestingly, when equality constraints were placed on the latent means the deterioration of CFI was more than the 0.01 (−0.017), a value which is typically used to support invariance constraints. On the other hand, however, the ΔRMSEA (0.012) was below the suggested cutoff value of 0.015 and substantially larger than in the other models. Obviously ΔCFI and ΔRMSEA values are not in line and lead to different conclusions about the existence of gender differences. However, based on prior literature on student-teacher relationships which suggests the existence of gender differences (e.g., Tsigilis and Gregoriadis, 2008; Solheim et al., 2012) and the way other authors handled similar situations (e.g., Guay et al., 2015) it was decided to proceed and interpret gender differences. Boys served as the reference group by fixing latent means to 0 for identification purposes and girls were the focal group by freely estimating them. Results showed statistically significant latent means on all SRTS subscales. In particular, girls displayed lower levels of conflict with their teachers than boys (*M* = −0.406, *SE* = 0.039, *p* < 0.001) and higher levels of closeness (*M* = 0.343, *SE* = 0.035, *p* < 0.001) and dependency (*M* = 0.311, *SE* = 0.037, *p* < 0.001).

To quantify the differences between boys and girls, Cohen's *d* was employed. Because the assumption of equal variances was satisfied, we used the common standard deviation for gender. Cohen's *d-*values were 0.41, 0.36, and 0.25 for conflict, closeness and dependency, respectively. Based on Cohen's guidelines for interpreting effect size values, the latent mean differences were small to moderate.

### STRS invariance across age

Invariance testing across age for the ESEM model provided similar results like the gender invariance. In particular, all increasingly restrictive modes yielded a satisfactory fit to the data based on the approximate fit indices. Changes in CFI and RMSEA were either trivial, suggesting no significant deterioration of the model or improvement indicating a better fit (Table 4). The only difference in relation to the gender invariance was noticed for the latent means. When equality constraints were imposed on the latent means, the fit of the model did not significantly worsen. Thus, the factorial structure of STRS can be regarded as invariant (measurement and structural) between preschoolers and early primary year's children.

**Table 4:** Summary of Goodness-of-Fit Statistics for the examined invariance across age.

	**χ^2^**	***df***	**CFI**	**RMSEA [90%CI]**	**Δχ^2^ (*df*)**	**ΔCFI**	**ΔRMSEA**
3–5 years old	2910.6	297	0.950	0.059 [0.057–0.061]	–	–	–
5–7 years old	1667.2	297	0.963	0.054 [0.052–0.057]	–	–	–
Configural Invariance	4530.3	594	0.955	0.057 [0.055–0.058]	–	–	–
Metric Invariance	2591.8	669	0.978	0.037 [0.036–0.039]	146.0 (75)^**^	+0.019	−0.016
Scalar Invariance	3079.7	778	0.974	0.038 [0.037–0.039]	612.0 (109)^**^	−0.004	+0.001
Variance-Covariance Invariance	2309.8	784	0.983	0.031 [0.029–0.032]	42.9 (6)^**^	+0.009	−0.007
Latent means invariance	2398.1	787	0.982	0.032 [0.030–0.033]	40.3 (3)^**^	−0.001	+0.001

** *p < 0.01*.

## Discussion

The present study was designed as a response to Drugli and Hjemdal's (2013) call for further research activity on the factorial validity of the STSR long form using a representative sample of school children. Findings are encouraging and seem to support the proposed three factor structure of the STRS. Our confidence is based on two strengths of the study. First, data were collected from a Greek representative sample, by applying a multistage sampling technique. This aspect of the design allows, for the first time in the Greek early childhood education setting, the generalizability of the results. Second, ESEM a methodologically sound and flexible statistical technique (Asparouhov and Muthén, 2009) was employed to examine the underlying structure of the STRS. ESEM is a very promising approach because it combines the advantages of both EFA and CFA into a single framework. Previous studies have shown that application of ESEM resulted in a superior model fit in comparison to CFA in various instruments in the field of social sciences (e.g., Marsh et al., 2010; Sánchex-Carracedo et al., 2012; Guay et al., 2015). This tendency was also present in our study. In particular, all goodness-of-fit indices yielded satisfactory values under the ESEM approach in contrast to the CFA analysis, which were unsatisfactory. This is the first version of the STRS that shows satisfactory fit by means of ESEM. Marsh et al. (2010) argue that unless items cross-loadings are close to zero, a CFA solution is similar to ESEM. In the present investigation, the obtained absolute values of several items cross-loadings were significant and of substantial size (e.g., items 5, 7, and 24). Forcing STRS items to load only on their respective latent factors seems to be an overly restricted assumption. Based on the above considerations, our suggestion is that ESEM can be used as a viable alternative of CFA in future studies about the quality of relationships between teachers and students.

The Closeness dimension is the one with the most pronounced cross-loadings to other STRS dimensions. Six out of eleven Closeness items had cross-loadings size above 0.30, and five of them were related to the Dependency subscale. This finding suggests the difficulty to develop items that capture solely the warm and positive relationships between teachers and students. It also shows the close conceptual linkage between Closeness and Dependency, at least in the Greek educational setting. On the contrary Conflict dimension appears to be well-understood and more precisely assessed.

It is worth noting that no modifications to the proposed model were introduced in order to improve its fit (e.g., discarding items, correlated items uniqueness). The only peculiar finding was that item 12 (“This child tries to please me”), originally designed to assess Closeness, had its principle loading on the Dependency. The problematic behavior of this item was also reported in previous studies conducted in Germany (Glüer and Gregoriadis, 2017) and Norway (Solheim et al., 2012). A possible explanation for the contradictory “behavior” of this item refers to its ambiguous content (Solheim et al., 2012). Solheim et al. (2012) suggested that the verb “to please” can be interpreted as something either positive (e.g., showing intention to cooperate and demonstrate prosocial behavior) or negative (e.g., something the child does to obtain an advantage or benefit). And in their occasion, they argued that Norwegian preschool teachers may have interpreted the item with its negative explanation. To take this argument one step further, the differentiated interpretation of the item could be attributed not only to the value and belief system of the teachers, but to the cultural influence of different cultural backgrounds. For example, previous studies (e.g., Gregoriadis and Tsigilis, 2008; Gregoriadis and Grammatikopoulos, 2014; Tsigilis et al., 2017) have shown that it is possible that Greek early childhood teachers interpret this item as an indicator of a child's more dependent behavior, which they don't necessary interpret as a negative characteristic.

Relative with this finding, the results from the inter-correlations among the three STRS factors, revealed once again a positive correlation between Closeness and Dependency. This contradictory finding that has been reported again in three previous studies in Greece (Gregoriadis and Tsigilis, 2008; Gregoriadis and Grammatikopoulos, 2014; Tsigilis et al., 2017) is in contrast with studies from other Western countries like for example the USA (Webb and Neurath-Pritchett, 2011), Netherlands (Koomen et al., 2012), Germany (Milatz et al., 2014; Glüer and Gregoriadis, 2017), Italy (Fraire et al., 2013), and Norway (Solheim et al., 2012). A possible explanation for this variation was attributed to cultural differences. Gregoriadis and Tsigilis (2008) suggested that dependency might be perceived differently in a collectivistic and in an individualistic environment. Past studies (Greenfield et al., 2003) have implied the existence of different cultural pathways in mother-child relationships. Thus, cultural influences on relationships can be considered an interesting issue that requires further examination. Especially for the construct of Dependency, “the meaning and interpretation of a dependent relationship may be subject to cultural differences” (Solheim et al., 2012, p. 260). The recurring finding of a positive association between Closeness and Dependency in three Greek studies and in the current study conducted in a Greek representative sample, allows the authors to express their certainty about the cultural influence on teacher-child relationships. Such a conclusion could also initiate a discussion whether the secure base concept is universal in social relations (van Ijzendoorn and Sagi, 2008; Milatz et al., 2014) or if cultural influences are a decisive factor for the quality of adult-child relationships. Future research efforts should examine in more depth this interesting issue.

Another purpose of the study was to examine the invariance of STRS across gender and age. These analyses could not have been conducted appropriately with the application of EFA, or with the CFA, given its low fit to the data. To the best of our knowledge this is the first attempt to study the structural invariance of the long version of STRS in addition to the measurement invariance. It is known that valid and meaningful latent factor means comparisons require strong invariance, partial or full. The present study demonstrated that STRS measurement invariance across students' gender holds, and subsequent latent factor means comparisons showed that teachers describe their relationships with girls as more affective and dependent and less conflictual than boys. In the Greek early childhood education settings, with few exceptions, the kindergarten teachers are female. This tendency was confirmed in our sample as well, in which only six teachers (1.12%) were male. Thus, the findings of this study about teacher-child relationship gender differences reflect mainly the female teachers' perceptions. According to Solheim et al. (2012) “female teachers find it easier to develop a close relationship to girls than boys because they identify more with the ways in which girls interact” (p. 260). This pattern of differences has been reported in various European countries including Netherlands (Koomen et al., 2012), Greece (Gregoriadis and Tsigilis, 2008), Italy (Fraire et al., 2013), and Norway (Solheim et al., 2012) with diverse contexts and irrespective of the statistical method used. As for the male teachers' perceptions, a recent study (Quaglia et al., 2013) showed that “…the male teacher's perception of the relationship is not influenced by the child's gender, unlike that of the female teacher, whose assessment of the relationship differs significantly according to the gender of the child” (p. 73). Finally, different effect of gender on teacher-child relationships was found in other studies, that however had relatively small sample sizes (e.g., Cadima et al., 2015; Glüer and Gregoriadis, 2017).

With regard to age, findings showed that STRS exhibits measurement and structural invariance. Thus, the form and meaning of teacher-child relationship quality in the Greek context seem to remain the same across the examined age span (3–6 years). Koomen et al. (2012) reported only metric invariance between two age subgroups (3–5.90 and 5.90–8.00 years old) of Dutch students. In another study, Milatz et al. (2014) found partial scalar invariance across kindergarten and 1st and 2nd grade of elementary German and Austrian schools. In both studies CFA procedures were applied to study the factorial structure of STRS and the invariance testing was based on large modifications of the instruments in order to reach reasonable fit indices. According to several authors invariance testing starts by establishing the best fitting model for each subgroup (Dimitrov, 2010; Byrne, 2012; Bowen and Masa, 2015; Pendergast et al., 2017). We feel that prior misspecified STRS models as conceptualized within the CFA framework, in which items cross-loadings were ignored, not only did they prevent authors from finding an acceptable fit, but also prohibited the establishment of the instrument's invariance. This discrepancy between our study and previous ones further demonstrates the usefulness of ESEM in testing the psychometric properties of an instrument.

This study of course is not free of limitations. First, research on EFA and CFA as methodological tools dates back several decades, whereas the ESEM is only recently developed. Thus the applied cut-off values for evaluating the fit of the examined models and group invariance in our study where developed in the CFA context using specific estimators, mostly the maximum likelihood method (e.g., Hu and Bentler, 1999). Assessment of a model's appropriateness and acceptance of the invariance might have been different if specific guidelines for ESEM had been proposed. Second, ESEM has its own limitations which are outlined in an excellent article by Marsh et al. (2014). One of these concerns the difficulty of fitting multilevel models. Clearly our data have an inherently hierarchical structure and a multilevel approach would have been more appropriate. At the moment only alternative approaches exist, in which the within- and between-covariance matrices are treated as separate sets of factors, or the ESEM within CFA approach (see Marsh et al., 2014 for details). However, these approaches have not been fully explored.

Aside from the above limitations, the present study replicated the proposed three-factor structure and supports the appropriateness of Greek version of STRS. Inherent shortcomings of EFA (e.g., incapacity to introduce residual error covariances) and CFA (excessively restrictive model) in prior studies were overcome by means of ESEM. Findings also maintain the generalizability of STRS factor structure across students' gender and age and provide the basis for meaningful means comparisons.

## Author contributions

The manuscript was prepared with the cooperation of all authors. All authors contributed to the study's research design. NT, AG, VG, and EZ contributed to the data collection procedure. All authors worked together for the writing and completion of the manuscript.

### Conflict of interest statement

The authors declare that the research was conducted in the absence of any commercial or financial relationships that could be construed as a potential conflict of interest.
